# Machine Learning for Predicting Colorectal Cancer‐Specific Mortality: The Role of Socioeconomic Inequalities in Public Policy

**DOI:** 10.1111/ans.70572

**Published:** 2026-03-18

**Authors:** Felipe Mendes Delpino, Rocco Friebel, Francisco Tustumi, Marina Martins Siqueira, Gabriely Rangel Pereira, Marcelo Passos Teivelis, Lucas Hernandes Correa, Nelson Wolosker

**Affiliations:** ^1^ Center for Studies and Promotion of Health Policies Hospital Israelita Albert Einstein São Paulo Brazil; ^2^ Department of Health Policy London School of Economic and Political Science London UK

**Keywords:** cancer‐specific mortality, colorectal cancer, machine learning, prediction

## Abstract

**Background:**

Colorectal cancer remains a leading cause of mortality worldwide. We investigated whether adding socioeconomic information to machine learning models can improve the prediction of colorectal cancer‐specific mortality.

**Methods:**

Using data from the *Fundação Oncocentro de São Paulo* (FOSP), we analyzed individuals diagnosed with colorectal cancer between 2000 and 2023; however, predictive models were developed using patients diagnosed from 2000 to 2021, ensuring a minimum follow‐up of 24 months for the 2‐year mortality outcome. Thirty predictor variables were included, including clinical factors associated with the disease and socioeconomic factors such as income, educational attainment, HDI components, as well as distance and travel time to healthcare facilities. We tested seven machine learning algorithms using a 70/30 training/testing split. Discrimination was measured by the area under the receiver operating characteristic curve (AUC‐ROC), comparing versions with and without socioeconomic factors.

**Results:**

The Random Forest algorithm provided the best discrimination for predicting the risk of death due to colorectal cancer within 2 years after diagnosis (AUC‐ROC = 0.92). The addition of socioeconomic and access‐related predictors (Human Development Index [HDI] components, education, distance/travel time to healthcare facilities, and type of coverage) improved the AUROC by 0.13 (0.79–0.92) compared with the clinical‐only model.

**Conclusion:**

The inclusion of socioeconomic variables in conjunction with clinical data in machine learning models has the potential to enhance the ability to predict colorectal cancer‐specific mortality in patients with colorectal cancer.

## 1 Introduction

Colorectal cancer is an urgent global health problem, ranking third among the most common cancers and second in terms of cancer mortality worldwide [1]. The year 2020 brought almost 1.9 million new diagnoses and more than 935 000 deaths worldwide [2], while in Brazil, the National Cancer Institute accounts for about 41 000 cases annually, making it a fundamental concern for health systems [1]. Advances in detection and therapy have been encouraging, but mortality rates remain high [2], paving the way for better ways to assess risks and direct resources to patients at higher risk of death.

In colorectal cancer, identifying mortality risks can be complex, requiring the separation of prognostic and predictive causes. Prognostic markers, such as TNM tumor staging, highlight patients who face higher probabilities from the outset and have been used for a long time in daily practice [3, 4]. On the other hand, predictive factors have the potential to identify who can benefit from personalized care or who encounters solvable obstacles along the way. Staging maintains its position as the core of prediction, but new perspectives indicate that socioeconomic elements can be important predictors both as risk signals and as starting points for more far‐reaching solutions at the community level [5].

The socioeconomic environment can significantly impact the paths taken by patients diagnosed with colorectal cancer. Patients residing in regions with low socioeconomic conditions, as measured by tools such as the Human Development Index (HDI) and basic measures of education and income, tend to avoid screening tests, present themselves at clinics at an advanced stage of the disease, fail to complete recommended follow‐up tests or treatments, and do not receive adequate supportive care [6, 7]. The unequal distribution of land and wealth in Brazil reinforces these inequalities; the state of São Paulo is an example of a place where HDI indices vary considerably between rich and poor areas. Social environments play a role in shaping risk profiles and the ability to manage and overcome the challenges posed by cancer [8, 9, 10]. Geographic distance and access to treatment centers represent broader barriers to timely detection and treatment, while educational attainment may influence understanding of symptoms and/or adherence to treatment recommendations. Although Brazil has implemented free public health services and paid private health services, there is still a lack of available research‐based data due to the complexities inherent in these systems.

Machine learning offers an approach with the potential to surpass classical statistical methods, as this approach can make predictions of patient mortality by taking into account the complexity of the relationships between variables [11, 12, 13]. Techniques such as Random Forest and Gradient Boosting have proven effective in predicting health‐related outcomes in different settings [11, 14, 15]. In this study, the objective was to fill the existing gap in the prediction of colorectal cancer‐specific mortality by developing and evaluating machine learning models using clinical data from the state of São Paulo, Brazil. The focus is on quantifying the improvement in model performance when incorporating socioeconomic variables (such as HDI component scores, geographic access to healthcare, and educational level), in addition to traditional clinical variables. In addition, we aim to identify the socioeconomic variables with the greatest socioeconomic impact that affect disease outcome and provide guidance for targeted interventions at the population level.

## 2 Methods

### 2.1 Study Design and Participants

We conducted a retrospective cohort study using data from the *São Paulo Oncocentro* Foundation (FOSP) database (http://www.fosp.saude.sp.gov.br), which includes cancer cases from 81 hospitals in the state of São Paulo, including both public and private hospitals. The database is open access and is generated by institutions in the state of São Paulo that, under the coordination of FOSP, maintain the Hospital Cancer Registry (RHC), forming the state RHC database. For this analysis, we included all cases of colorectal cancer recorded in the database from 2000 to 2023.

### 2.2 Outcome

The primary outcome was colorectal cancer‐specific mortality within 2 years of diagnosis. Mortality data were obtained from the RHC/SP (FOSP Hospital Cancer Registry), with the underlying cause of death identified from death certificates. Only deaths in which colorectal cancer was recorded as the underlying cause were considered events.

Deaths attributed to causes other than colorectal cancer (e.g., accidents, homicide, or suicide) were excluded from the analytical cohort prior to model development to maintain a cause‐specific prediction framework. Accordingly, the binary outcome distinguished between colorectal cancer‐specific death and survival within the fixed 24‐month horizon.

Although deaths from other causes represent competing events, the objective of this study was prediction of colorectal cancer‐specific mortality at a fixed time point rather than estimation of cause‐specific hazards. Therefore, competing‐risk methods were not applied. This approach was considered appropriate for the predictive objective, although some residual bias related to competing mortality cannot be fully excluded.

Patients diagnosed in 2022 or later were included only in descriptive analyses due to insufficient follow‐up for the 2‐year outcome. Thus, the primary models predicted the risk of colorectal cancer‐specific mortality at 2 years after diagnosis among patients diagnosed between 2000 and 2021. Additionally, time‐stratified models were estimated: the ≤ 2‐year model used the fixed 2‐year outcome, whereas the > 2‐year model evaluated mortality beyond 2 years among patients who survived at least 2 years.

### 2.3 Follow‐Up

We calculated follow‐up from the date of colorectal cancer diagnosis (index date) until colorectal cancer‐specific death. Patients who did not experience colorectal cancer‐specific death within 24 months were classified as nonevents. We excluded deaths attributed to causes other than colorectal cancer from the analytical cohort prior to model development to preserve the cause‐specific prediction framework.

The primary outcome was colorectal cancer‐specific mortality within 24 months after diagnosis. We restricted model development to patients diagnosed between 2000 and 2021 to ensure complete 2‐year follow‐up; patients diagnosed from 2022 onward were included only in descriptive analyses. We adopted a fixed 24‐month horizon to standardize outcome definition and minimize follow‐up‐related bias in the binary classification models.

### 2.4 Predictors

Based on consultation with clinical oncologists and colorectal surgeons, we selected 30 predictor variables (see Table S1 for the full list of predictor variables and their description) that included both the patient's cancer‐related clinical characteristics and sociodemographic characteristics. We enhanced the dataset by collecting the three indices that compose the HDI, income, education, and longevity, for each patient's municipality of residence. Additionally, we incorporated data on the road distance to the healthcare facility as a proxy for accessibility and the travel duration to reach the treatment location. We aimed to identify whether, in addition to the patient's clinical characteristics, sociodemographic characteristics would be predictors of death.

### 2.5 Algorithms

We tested seven algorithms commonly used in clinical prediction studies, namely: Random Forest, Gradient Boosting, XGBoost, LightGBM, CatBoost, Logistic Regression, and Decision Trees. Each algorithm was trained and tested to predict colorectal cancer‐specific mortality based on the predictors mentioned in the previous subtopic [11, 16]. To quantify the added value of socioeconomic variables, we trained paired models: one model with clinical variables only, and an extended model including socioeconomic and access variables (education, type of health coverage, municipal HDI components, distance, and travel time).

### 2.6 Preprocessing

The dataset was divided into training (70%) and testing (30%) using stratified sampling to preserve the proportion of events (colorectal cancer‐specific mortality versus survival) in both divisions. All preprocessing steps were performed exclusively on the training data to avoid information leakage and were subsequently applied without changes to the reserved test set. Continuous predictors were normalized, and categorical predictors were encoded using one‐hot encoding.

To reduce redundancy and improve model stability, multicollinearity among predictors was assessed using the variance inflation factor (VIF). VIF‐based variable exclusion was applied only to logistic regression models and not to tree‐based algorithms, for which multicollinearity is not a methodological concern. To address class imbalance, RandomUnderSampler was applied exclusively to the training set [17]. In the analytical cohort, colorectal cancer‐specific mortality occurred in 27 535 of 62 485 patients (44.1%), indicating moderate class imbalance. Subsampling was implemented conservatively, resulting in a balanced 1:1 ratio in the training set (19 274 events and 19 274 nonevents), while the original distribution of events was preserved in the reserved test set.

### 2.7 Missing Data and Exclusion Criteria

We evaluated the missing data among the variables included in the predictive models (Table S1). Exclusions were motivated exclusively by the lack of information in essential model variables related to sociodemographic aspects, care trajectory, and health system organization (EDUCATION, ATTENDANCE_CATEGORY, DIAGTRAT, and TRATCONS), resulting in the exclusion of 4631 individuals (6%). The patterns of missing data were examined overall and stratified by outcome (death vs. survival), and no relevant differences were found. Given the low proportion of exclusions and the sample size, we chose to adopt a complete case analysis and did not perform multiple imputation.

### 2.8 Hyperparameter Tuning

The search for hyperparameters was optimized using a randomized search algorithm (RandomizedSearchCV) and applied only to the training data. For each algorithm included in this study, we performed 5‐fold cross‐validation on 20 randomly generated combinations of hyperparameters and selected the configuration that achieved the highest area under the ROC curve (AUC‐ROC). The test set was never used during hyperparameter tuning or model selection. Each of the selected models with the best performance was then retrained from scratch using the entire training dataset.

### 2.9 Performance Metrics

The performance of the models was evaluated in the test set using standard classification metrics, including the area under the ROC curve (AUC‐ROC), accuracy, precision, sensitivity (recall), specificity, Matthews correlation coefficient (MCC), and F1‐score. The main metric adopted was AUC‐ROC, with values ≥ 0.70 being considered acceptable for discrimination. Given the presence of moderate class imbalance and the clinical relevance of sensitivity, the classification decision threshold was selected in the training set by maximizing the F1 score and subsequently applied unchanged to the test set. All analyses were conducted in Python using a Google Colab environment.

To support model interpretability and identify the most influential predictors, we applied SHAP (Shapley Additive Explanations) [18]. SHAP values quantify each feature's contribution to individual predictions, and SHAP summary plots were used to rank variables by global importance and to illustrate the direction and magnitude of their effects (e.g., age and cancer stage).

### 2.10 Additional Analysis

For comparison purposes, we tested an additional model excluding socioeconomic and access‐related variables (HDI components, education, distance to care, and travel time). A Kaplan–Meier survival curve was also created to assess the survival in months of the participants. Based on this, we created two additional models to evaluate the predictive performance of the models: the first for deaths that occurred before 2 years and the second after 2 years or more. This analysis was carried out to understand how the models perform when mortality occurs over a relatively longer time.

### 2.11 Reproducibility

For transparency and reproducibility, the final hyperparameters of all machine learning models after cross‐validated optimization are reported in Table S3. Model training and evaluation followed a standardized pipeline, including class balancing, cross‐validation, and threshold optimization.

## 3 Results

### 3.1 Participant Selection and Flow

**FIGURE 1:**
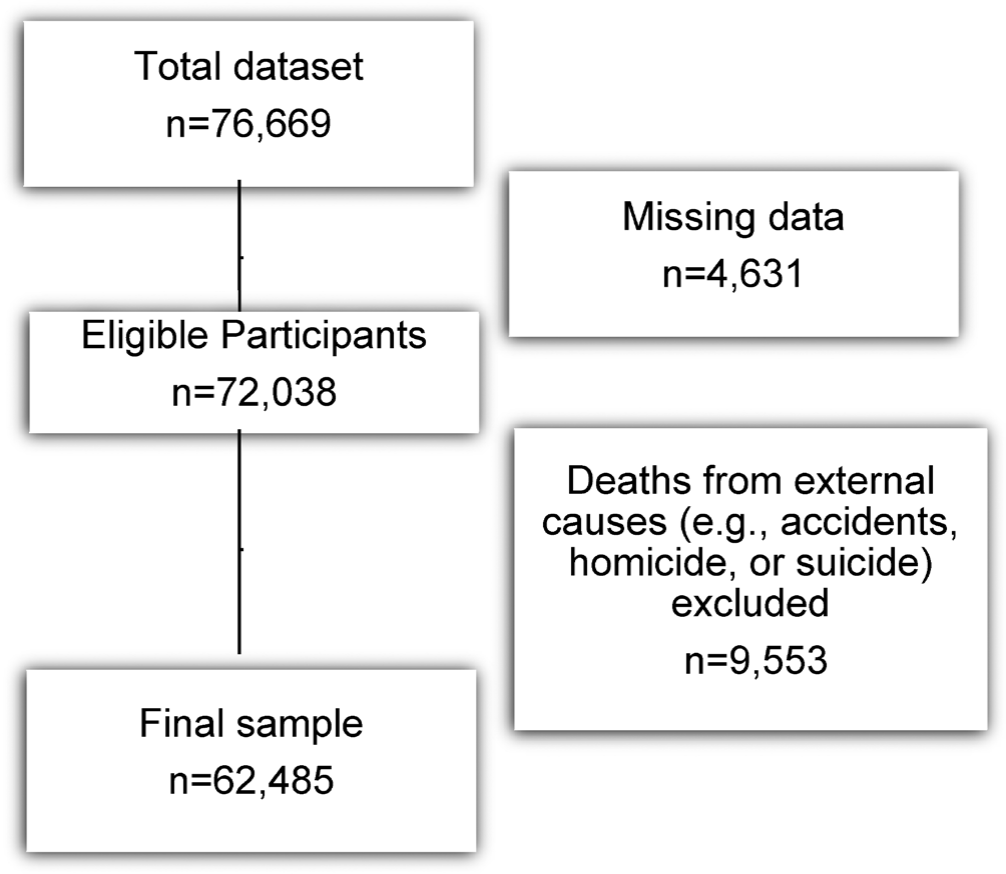
Flow chart describing sampling process.

Figure 1 illustrates the participant selection process in the present study. The initial sample consisted of 76 669 patients diagnosed with colorectal cancer, as recorded in the FOSP registry. The exclusion of 4631 individuals (6.0%) occurred due to insufficient information on essential analytical variables, resulting in 72 038 eligible cases. Subsequently, 9553 records (12.5%) were excluded in which the recorded cause of death was attributed to external factors, such as accidents, homicide, or suicide. This resulted in a final analytical cohort of 62 485 patients, representing 81.5% of the original study population.

### 3.2 Sociodemographic Characteristics

Table 1 shows the sociodemographic characteristics of the study population, categorized based on survival status at the end of follow‐up. Among those who died, men were more frequent (53.6%, 95% CI: 53.0–54.2) than women (46.4%, 95% CI: 45.8–47.0), while survivors had a balanced distribution between the sexes. The mean age at diagnosis was higher among those who died (mean 62.4 years, 95% CI: 62.3–62.6) than among survivors (mean 60.3 years, 95% CI: 60.1–60.4). There was a significant variation in educational level. Deceased individuals had a higher proportion of illiteracy (7.3%, 95% CI: 6.9–7.7) or complete elementary school education (41.5%, 95% CI: 40.8–42.2). On the other hand, survivors had higher rates of high school or higher education. Most deaths occurred among those who received treatment through the public health system (SUS), accounting for 91.9% (95% CI: 91.5–92.3) of deaths, while private insurance was more prevalent among survivors (17.0%, 95% CI: 16.6–17.5). The average Municipal Human Development Index (MHDI) for both groups was 0.78, with no significant differences observed. The median follow‐up time was 2.32 years (interquartile range: 0.79–5.45), with a maximum follow‐up time of 22.86 years.

**TABLE 1:** Description of individuals' sociodemographic variables.

Variables	*N* survival	Survival	*N* death	Death
Sex				
Male	17 192	49.2 48.7–49.7	14 764	53.6 53.0–54.2
Female	17 758	50.8 50.3–51.3	12 771	46.4 45.8–47.0
Mean age	—	60.3 60.1–60.4	—	62.4 62.3–62.6
Education				
Illiterate	1003	3.9 3.7–4.2	1473	7.3 6.9–7.7
Incomplete elementary education	9353	36.6 36.0–37.2	8383	41.5 40.8–42.2
Complete elementary education	5663	22.1 21.6–22.7	4994	24.7 24.1–25.3
High school	5782	22.6 22.1–23.1	3722	18.4 17.9–19.0
Higher education	3791	14.8 14.4–15.3	1633	8.1 7.7–8.5
Municipal Human Development Index (MHDI)	—	0.78 0.78–0.78	—	0.78 0.78–0.78
Service category				
Health insurance	4704	17.0 16.6–17.5	1240	7.6 7.2–8.0
Unified health system (SUS in Portuguese)	22 403	81.2 80.7–81.6	14 961	91.9 91.5–92.3
Private	491	1.8 1.6–1.9	73	0.5 0.4–0.6

*Note:* Values are proportion or mean with 95% of confidence interval.

### 3.3 Survival Analysis

**FIGURE 2:**
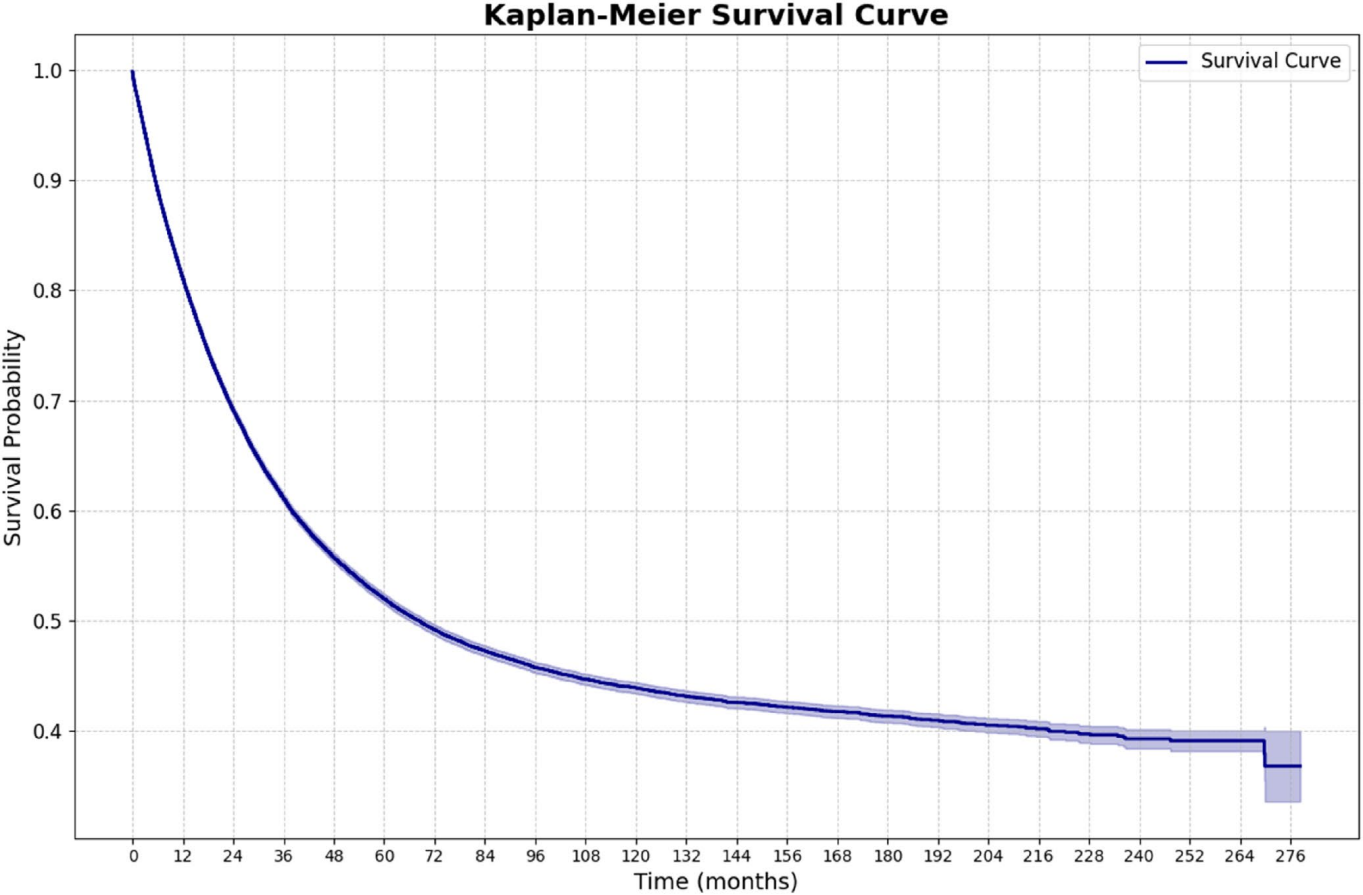
Kaplan–Meier cancer‐specific survival curve illustrating the survival probability over time (in months) for colorectal cancer patients. The blue line represents the estimated survival curve, while the shaded area indicates the 95% confidence interval. The curve shows a sharp decline in survival probability during the initial months, stabilizing around 35%–40% after approximately 10 years.

Figure 2 shows cancer‐specific survival estimates using the Kaplan–Meier methodology over a period of 23 years (276 months). The initial survival probability of 100% declined sharply in the first 2 years after diagnosis. Subsequently, the curve stabilized, indicating a reduction in risk in subsequent years. At the end of the study period, long‐term survival rates stabilized at around 35%–40%. The reduction in confidence intervals over time reflected greater accuracy in survival estimates as the number of patients at risk decreased.

### 3.4 Model Performance

**FIGURE 3:**
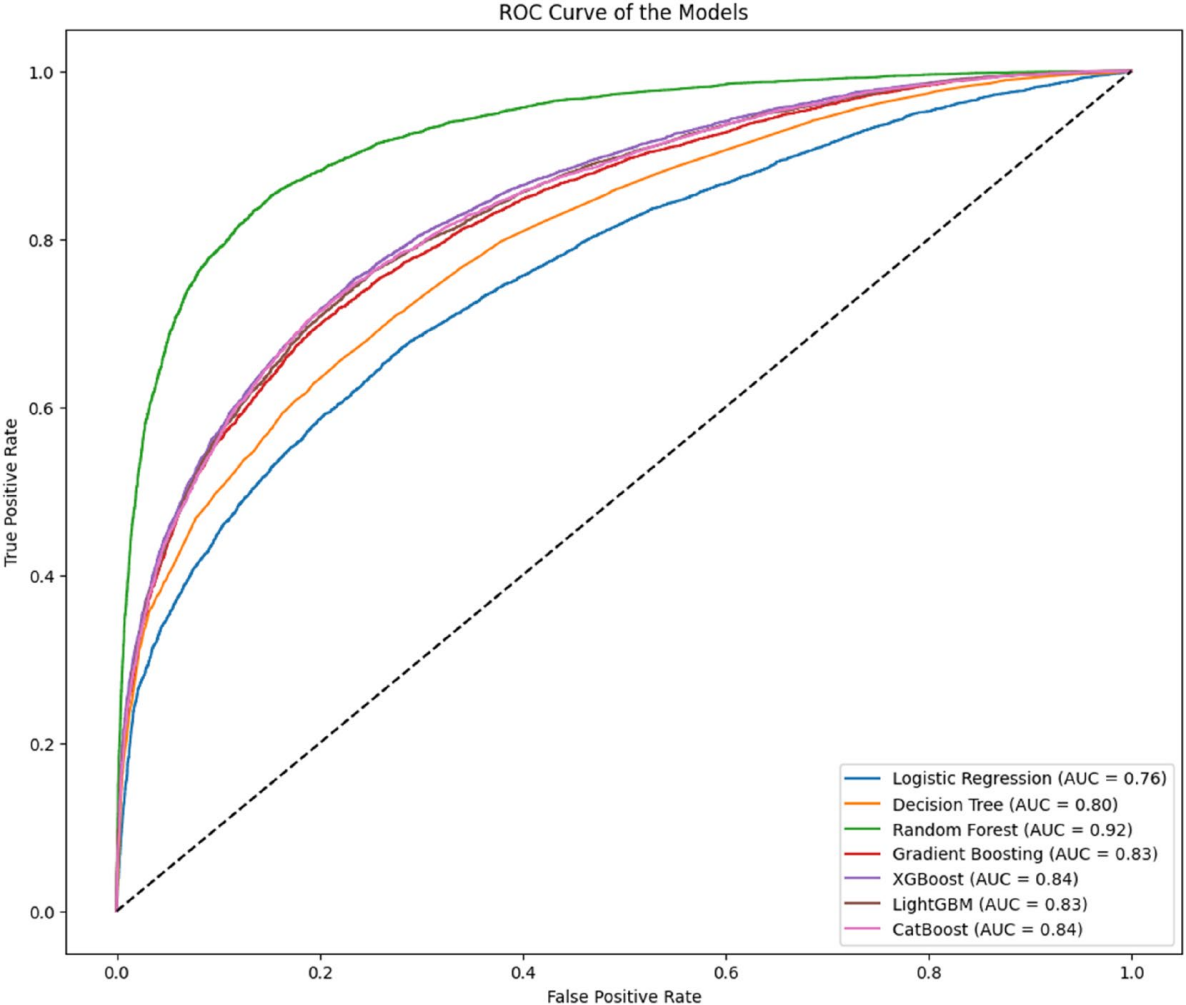
Roc curve showing the AUC value for the seven models on the test dataset.

Assessment of predictive models showed that Random Forest achieved the highest discriminatory performance for mortality prediction, with an AUC‐ROC of 0.92 (Figure 3). XGBoost and CatBoost demonstrated reasonable discrimination (AUC = 0.84) but were outperformed by Random Forest. When socioeconomic variables, including HDI components, education, and distance to care, were added to clinical variables, model discrimination increased by 13 percentage points compared with the clinical‐only model (Figure S1). The corresponding confusion matrix and feature importance for the clinical‐only model are presented in Figures S2 and S3.

As shown in Table 2, the Random Forest model achieved an overall accuracy of 85%, with a precision of 83%, sensitivity of 82%, and an F1‐score of 83%. Specificity increased from 0.80 in the clinical‐only model to 0.87 with the inclusion of socioeconomic variables (Table S2). The MCC for the Random Forest model was 0.70. In contrast, Logistic Regression showed the lowest performance, with an AUC of 0.76 and an MCC of 0.39.

**TABLE 2:** Models used and test results to predict cancer‐specific mortality in colorectal patients.

Models	Accuracy	Precision	Recall	F1‐score	Specificity	AUC	MCC
Random forest	0.85	0.83	0.82	0.83	0.87	0.92	0.70
XGBoost	0.76	0.73	0.73	0.73	0.79	0.84	0.52
CatBoost	0.76	0.73	0.73	0.73	0.79	0.84	0.51
LightGBM	0.76	0.72	0.73	0.73	0.78	0.83	0.51
Gradient boosting	0.75	0.72	0.72	0.72	0.78	0.83	0.50
Decision tree	0.73	0.70	0.64	0.67	0.79	0.80	0.44
Logistic regression	0.70	0.67	0.62	0.65	0.77	0.76	0.39

### 3.5 Confusion Matrix Analysis

**FIGURE 4:**
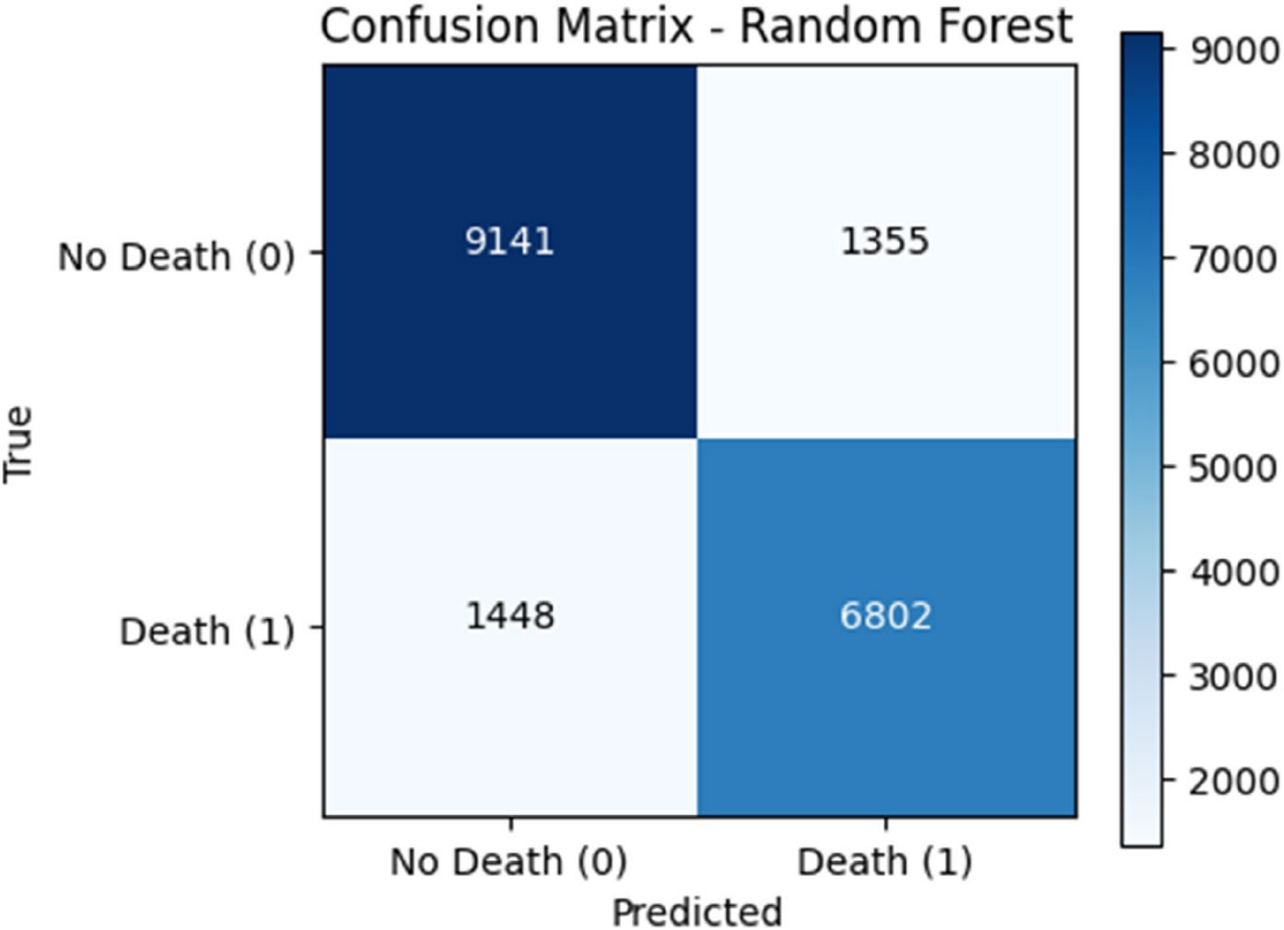
Confusion matrix for random forest model on the test dataset.

Figure 4 details the Random Forest confusion matrix, which included 9141 true negatives and 6802 true positives, along with 1448 false negatives and 1355 false positives. Specificity was high at 87%, allowing for reliable exclusion of nonevents, and recall reached 82%.

### 3.6 Feature Importance

**FIGURE 5:**
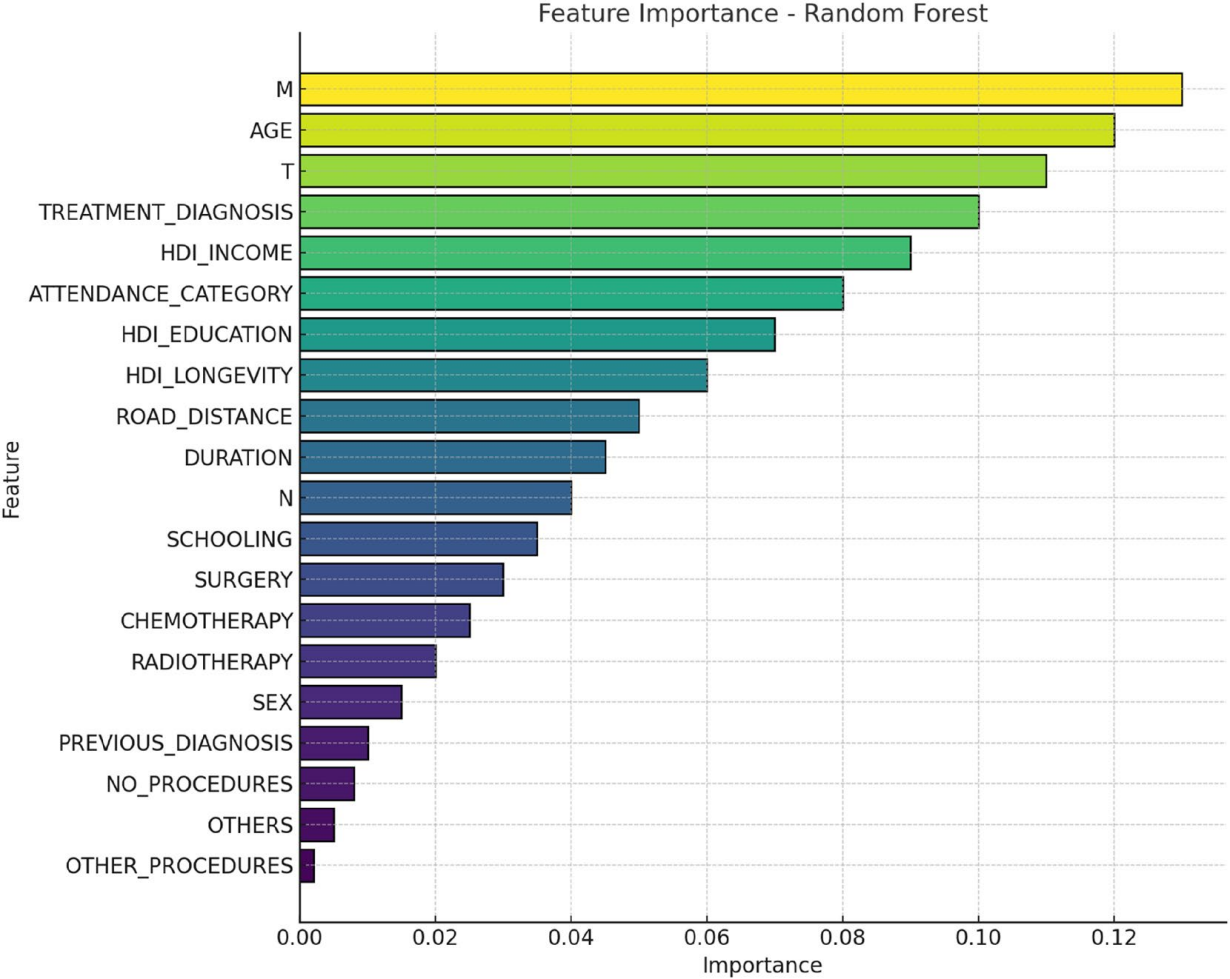
SHAP (Shapley Additive Explanations) summary plot showing global feature importance for the best‐performing model predicting 2‐year colorectal cancer‐specific mortality.

The use of SHAP values in Figure 5 showed that the mortality predictor with the greatest impact was cancer stage (M), followed by patient age and cancer stage (T). Other factors relevant to prediction included the time elapsed between diagnosis and treatment, municipal socioeconomic indicators (the three components of the HDI: income, education, and longevity), and the patient's health insurance coverage. In addition, predictors related to access (distance to the hospital and duration of treatment) also contributed to risk stratification. On the other hand, treatment‐related predictors (surgery, chemotherapy, and radiotherapy) were of relatively less overall importance. Given the relevance of socioeconomic and access‐related predictors, we complemented the SHAP summary plot with additional SHAP analyses in the horizon‐stratified models (Figures S6 and S9), illustrating how the set of influential predictors changes according to the time window of mortality.

### 3.7 Time‐Stratified Performance

When stratifying by time after diagnosis, Random Forest demonstrated higher performance for predicting early mortality (≤ 2 years), with an AUC of 0.95 (Figure S4), and health coverage emerging as the most influential predictor (Figures S5 and S6). For deaths occurring after 2 years, predictive performance decreased (AUC = 0.80; Figure S7), with M stage retaining predominant importance (Figures S8 and S9).

## 4 Discussion

The findings of this study demonstrate that incorporating socioeconomic data into machine learning models may improve the prediction of colorectal cancer mortality, achieving a potential increase of 13 percentage points in discriminatory power (AUC 0.92 vs. 0.79) compared to clinical variables when evaluated in isolation. These findings confirm our main premise that socioeconomic factors are associated with cancer mortality in Brazil, going beyond conventional clinical staging. The Random Forest model demonstrated robust overall performance, with balanced sensitivity (82%) and specificity (87%), facilitating the identification of high‐risk patients who could benefit from improved therapies.

Our results suggest that socioeconomic and access‐related factors may contribute to risk stratification; however, this is an observational study, and it is not possible to establish causal mechanisms. Geographic accessibility metrics (road distance and travel time) may be linked to higher mortality, as patients who face long travel times have increased mortality rates. This may be related to delays in diagnosis, treatment initiation, and subsequent care. The type of health coverage (public SUS versus private insurance) was a significant predictor of early mortality, indicating disparities in access to rapid, high‐quality care. The components of the municipal HDI (income, education, life expectancy) predicted outcomes independently. This may suggest that socioeconomic conditions at the neighborhood level influence health outcomes through factors such as local health infrastructure, environmental conditions, social support networks, and health promotion resources. In addition, the patient's level of education was also an important predictor, independent of clinical variables and the community‐level HDI [19]. This association may be mediated by health literacy, which can influence symptom recognition, treatment adherence, and engagement with preventive care. Significantly, these socioeconomic factors had a substantial impact on early death (≤ 2 years), while late mortality was mainly influenced by tumor biology (M stage).

The model's positive predictive value of 83% for mortality makes it possible to identify high‐risk patients who are more likely to benefit from support programs to overcome barriers to health equity, including geographical and bureaucratic obstacles. Furthermore, although our model identifies at‐risk populations, health systems have limitations in their ability to change structural variables, such as a municipality's HDI. Our model is designed to be used as an aid in the development of public policies aimed at reducing inequalities, rather than in individual clinical decision‐making. Decision‐makers can use the findings to provide screening programs, treatments, and support services to communities with low HDI and limited access to healthcare. In practice, the successful implementation of the model will depend on the risk threshold selected by the user, which will determine the balance between false positives and false negatives. Changing this threshold can result in substantially different resource use, referral rates, and subsequent clinical workload when used for patient screening or for prioritizing patients for screening or treatment. In addition to the above considerations, there is also concern that models developed based on historical health data may actually reinforce existing inequalities in healthcare delivery if differences in patterns of access, diagnosis, and treatment are reflected in the data used to train the model. Therefore, subgroup‐level analyses (e.g., sex, age, and tumor stage) and continuous monitoring of model accuracy and fairness are essential before implementation in a real‐world setting.

Our results build upon the previous research conducted by Buk Cardoso et al. [19], who documented an AUC of 0.86 utilizing the FOSP database (2000–2021) with clinical factors. Our enhanced performance (AUC 0.92) may be explained by the integration of socioeconomic predictors, assessment of a wider array of algorithms, incorporation of more recent data (up to 2023), and methodical comparison between models with and without socioeconomic factors. These analytical advancements can facilitate the quantification of the precise impact of socioeconomic variables on mortality prediction. Previous studies have established correlations between socioeconomic determinants, including HDI, and colon cancer outcomes [20, 21, 22]. Our study highlights that socioeconomic variables continue to be significant in a mixed healthcare system with universal public coverage, indicating that formal insurance access does not eradicate socioeconomic disparities in outcomes. Prior research has established correlations between HDI and many cancer forms in Brazil. Other studies demonstrated favorable outcomes using Random Forest models in health predictions [22, 23, 24], underscoring the usefulness of this approach for clinical prediction.

Some limitations are worth noting. Although extensive, the FOSP database does not include all cases of colorectal cancer in the state of São Paulo due to the voluntary nature of participation in the hospital registry. This may result in underrepresentation of patients treated in smaller health facilities or in rural areas. Second, the omission of deaths from other causes may increase selection bias, since mortality from causes unrelated to cancer may correlate with age and comorbidities. Future studies should use competing risk models to minimize this limitation. Third, our socioeconomic metrics predominantly reflect municipal and individual educational factors; the inclusion of other variables, such as family income, professional status, and neighborhood resources, could improve predictive accuracy. However, these variables are not available in the FOSP database. Fourth, external validation in other Brazilian states or other countries is necessary to assess the generalization of the model. It is important to note that this work should be interpreted as an exploratory prediction study based on retrospective data. Although the model's discrimination was high in internal tests, the model has not been externally validated, and we did not perform an impact assessment to evaluate clinical outcomes, feasibility, or cost‐effectiveness. Therefore, any operational use to inform public policy would require additional work, including external validation on independent datasets, calibration assessment, and prospective evaluation of benefits and unintended consequences. Finally, given the long study period, temporal changes in diagnostic practices, treatment availability, and healthcare organization may have occurred and could potentially affect model performance over time (concept drift). To mitigate this, models were trained and evaluated using data spanning the entire period, and performance was assessed on a temporally mixed test set. Nevertheless, formal temporal validation was beyond the scope of this study, and future work should evaluate model stability and recalibration across different calendar periods.

This study contributes to the field by demonstrating that machine learning models that incorporate socioeconomic data provide better mortality predictions than those based solely on clinical characteristics. Future research should focus on implementation science, attempting to identify how these advanced prediction models can be incorporated into clinical workflows and public health strategies to achieve improvements in outcomes with a focus on equity.

## 5 Conclusion

Our results showed how combining clinical data and social determinants of health through machine learning can be used to make decisions that help support the health and well‐being of individuals at risk for poor health outcomes due to colorectal cancer. The findings highlight the need for public health policymakers to consider the impact of socioeconomic disparities in the development of policies and interventions for colorectal cancer. To better address these disparities, policymakers can use the findings as an example of how they can work to expand access to quality healthcare in socioeconomic disadvantaged communities. This may include increasing access to primary care services, improving screening protocols, and establishing programs that allow them to target resources to specific geographic locations, such as counties, with low HDI scores.

## Author Contributions

**Felipe Mendes Delpino:** conceptualization, investigation, writing – original draft, methodology, writing – review and editing, formal analysis, project administration, validation, and visualization. **Rocco Friebel:** writing – review and editing. **Francisco Tustumi:** writing – review and editing. **Marina Martins Siqueira:** writing – review and editing. **Gabriely Rangel Pereira:** writing – review and editing. **Marcelo Passos Teivelis:** writing – review and editing. **Lucas Hernandes Correa:** writing – review and editing. **Nelson Wolosker:** writing – review and editing.

## Funding

This study was supported by the Bracell Foundation through two fellowship grants.

## Ethics Statement

This study used secondary data from the *Fundação Oncocentro de São Paulo* (FOSP) Hospital Cancer Registry, which are publicly available and de‐identified at the individual level. The study did not involve direct contact with participants and did not include identifiable personal information. All analyses were conducted in accordance with the registry's governance rules and terms of use; therefore, approval by a research ethics committee was not required.

## Conflicts of Interest

The authors declare no conflicts of interest.

## Supporting information


**Figure S1:** Roc curve showing the AUC value in the model without the socioeconomic variables.
**Figure S2:** Confusion matrix for random forest in the model without the socioeconomic variables.
**Figure S3:** SHAP graph showing the most important predictor variables in the model without the socioeconomic variables.
**Figure S4:** Roc curve showing the AUC value in the model considering colorectal cancer‐specific mortality in ≤ 2 years.
**Figure S5:** Confusion matrix for random forest in the model considering colorectal cancer‐specific mortality in ≤ 2 years.
**Figure S6:** SHAP graph showing the most important predictor variables in the model considering colorectal cancer‐specific mortality in ≤ 2 years.
**Figure S7:** Roc curve showing the AUC value in the model considering colorectal cancer‐specific mortality in > 2 years.
**Figure S8:** Confusion matrix for random forest in the model considering colorectal cancer‐specific mortality in > 2 years.
**Figure S9:** SHAP graph showing the most important predictor variables in the model considering colorectal cancer‐specific mortality in > 2 years.
**Table S1:** List of predictor variables used.
**Table S2:** Comparative performance metrics of random forest models with and without socioeconomic variables.
**Table S3:** Final hyperparameters of the machine learning models.

## Data Availability

The data that support the findings of this study are available from the corresponding author upon reasonable request.
