# Supplementary material for: Machine Learning for Predicting Colorectal Cancer‐Specific Mortality: The Role of Socioeconomic Inequalities in Public Policy
Source: ANZ J Surg. 2026 Mar 18;96(6):1717–26. doi: 10.1111/ans.70572 (PMC13327648; doi:10.1111/ans.70572)
Supplement: Supplementary file 1 — Figure S1: Roc curve showing the AUC value in the model without the socioeconomic variables. Figure S2: Confusion matrix for random forest in the model without the socioeconomic variables. Figure S3: SHAP graph showing the most important predictor variables in the model without the socioeconomic variables. Figure S4: Roc curve showing the AUC value in the model considering colorectal cancer‐specific mortality in ≤ 2 years. Figure S5: Confusion matrix for random forest in the model considering colorectal cancer‐specific mortality in ≤ 2 years. Figure S6: SHAP graph showing the most important predictor variables in the model considering colorectal cancer‐specific mortality in ≤ 2 years. Figure S7: Roc curve showing the AUC value in the model considering colorectal cancer‐specific mortality in > 2 years. Figure S8: Confusion matrix for random forest in the model considering colorectal cancer‐specific mortality in > 2 years. Figure S9: SHAP graph showing the most important predictor variables in the model considering colorectal cancer‐specific mortality in > 2 years. Table S1: List of predictor variables used. Table S2: Comparative performance metrics of random forest models with and without socioeconomic variables. Table S3: Final hyperparameters of the machine learning models. [file ANS-96-1717-s001.docx]

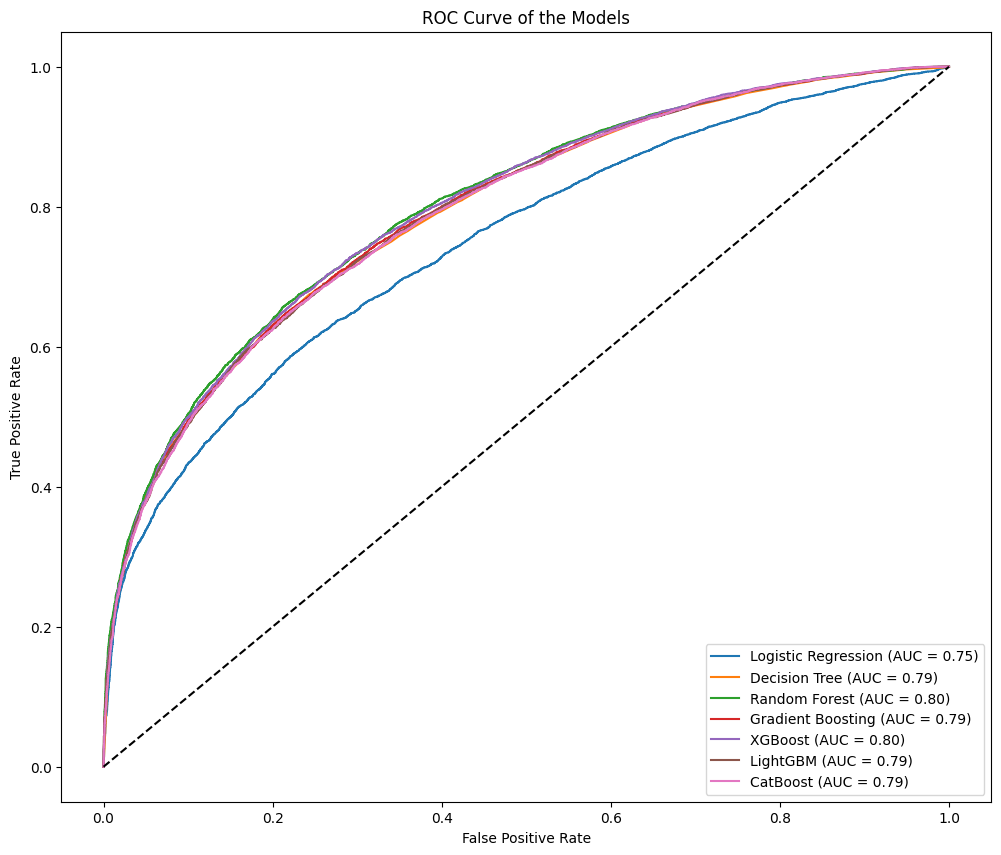


**Supplementary Figure 1**. Roc curve showing the AUC value in the model without the socioeconomic variables


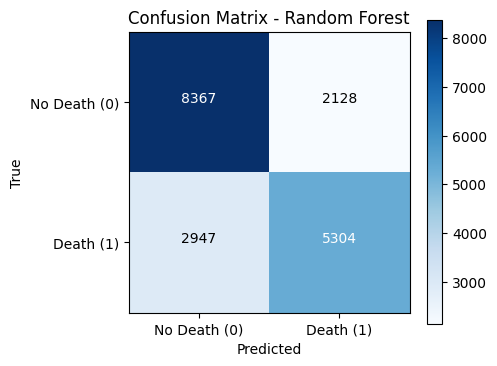


**Supplementary Figure 2**. Confusion matrix for Random Forest in the model without the socioeconomic variables


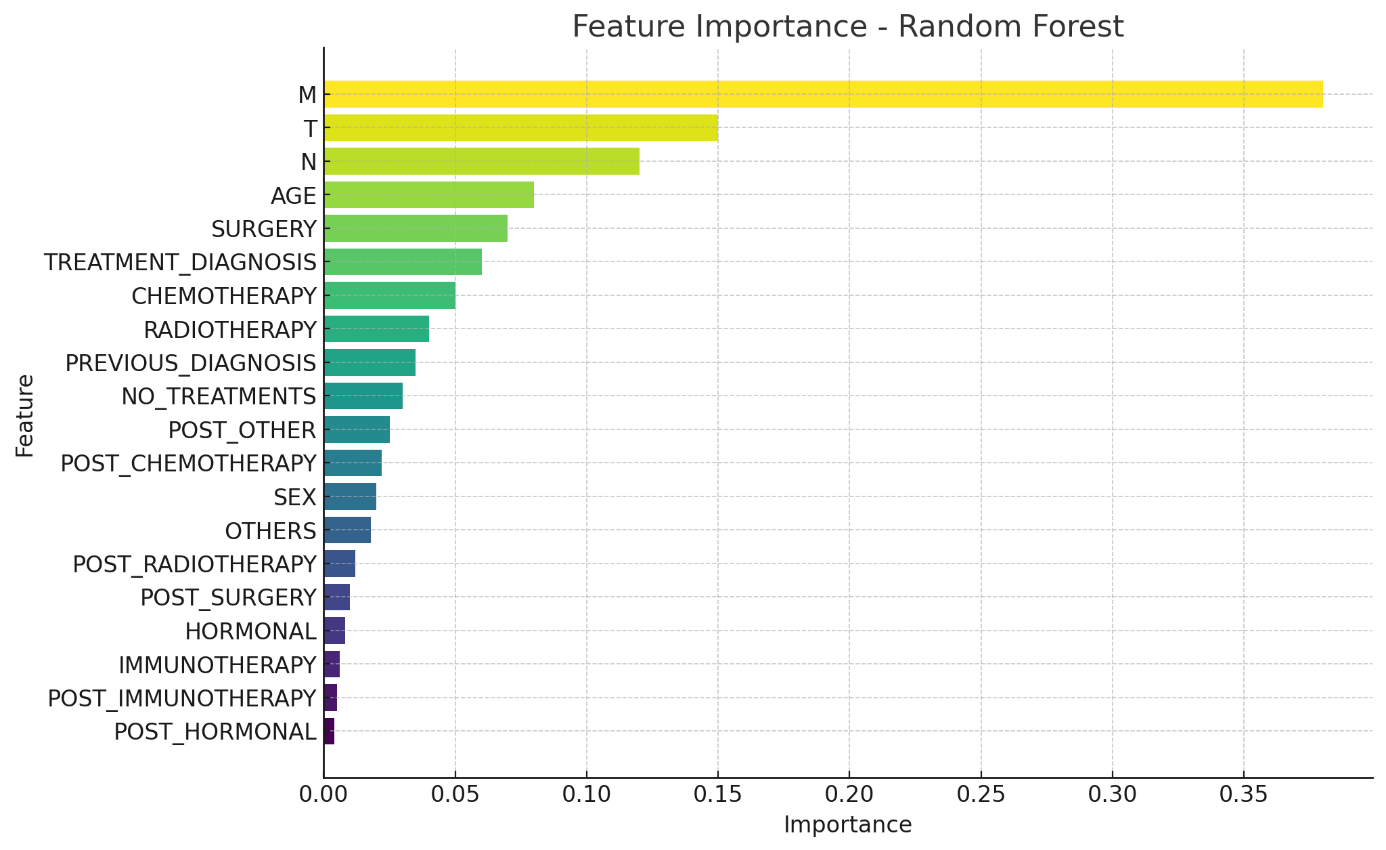


**Supplementary Figure 3**. SHAP graph showing the most important predictor variables in the model without the socioeconomic variables


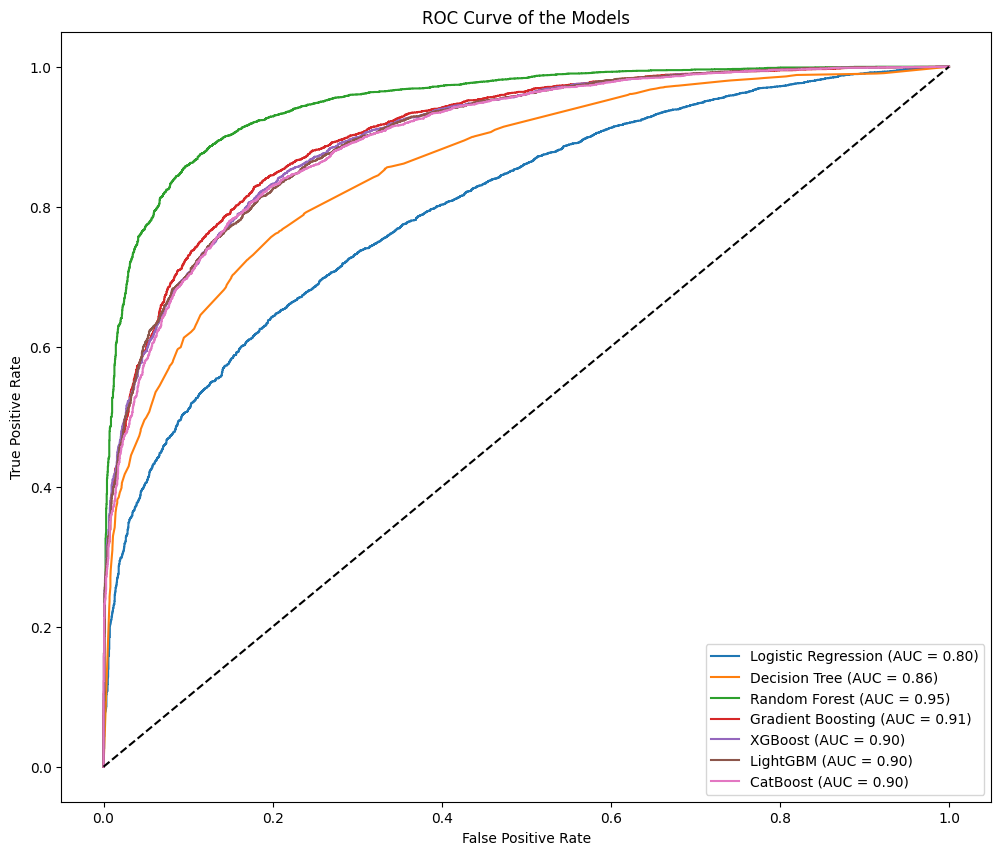


**Supplementary Figure 4**. Roc curve showing the AUC value in the model considering colorectal cancer-specific mortality in < 2 years


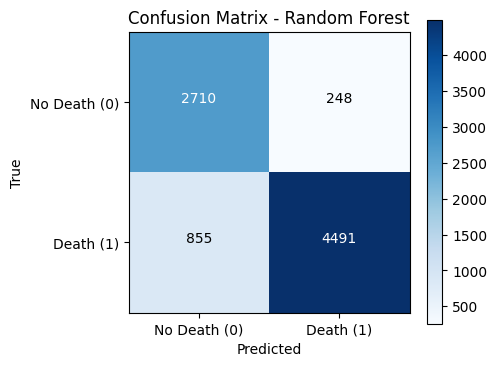


**Supplementary Figure 5**. Confusion matrix for Random Forest in the model considering colorectal cancer-specific mortality in < 2 years


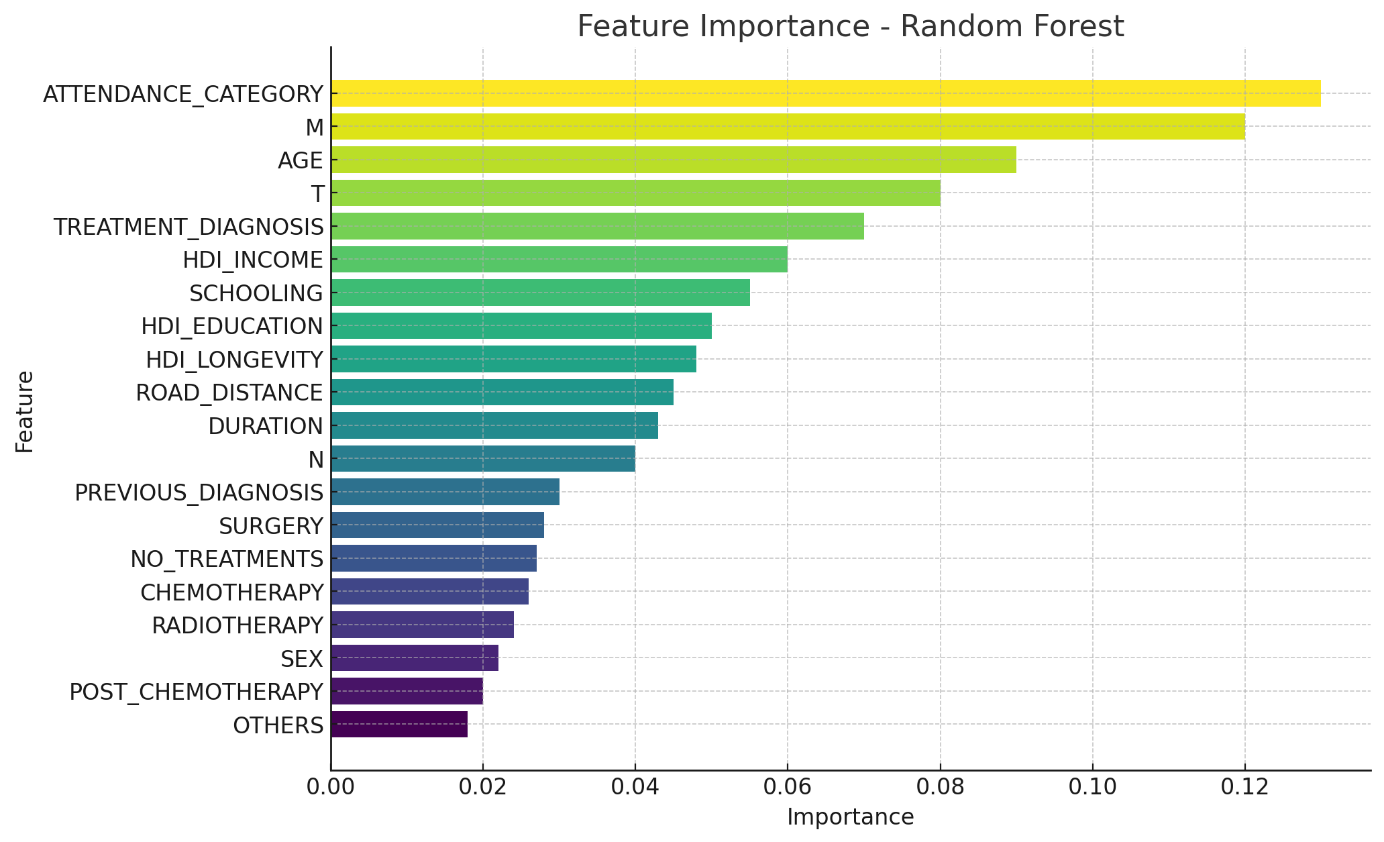


**Supplementary Figure 6**. SHAP graph showing the most important predictor variables in the model considering colorectal cancer-specific mortality in < 2 years


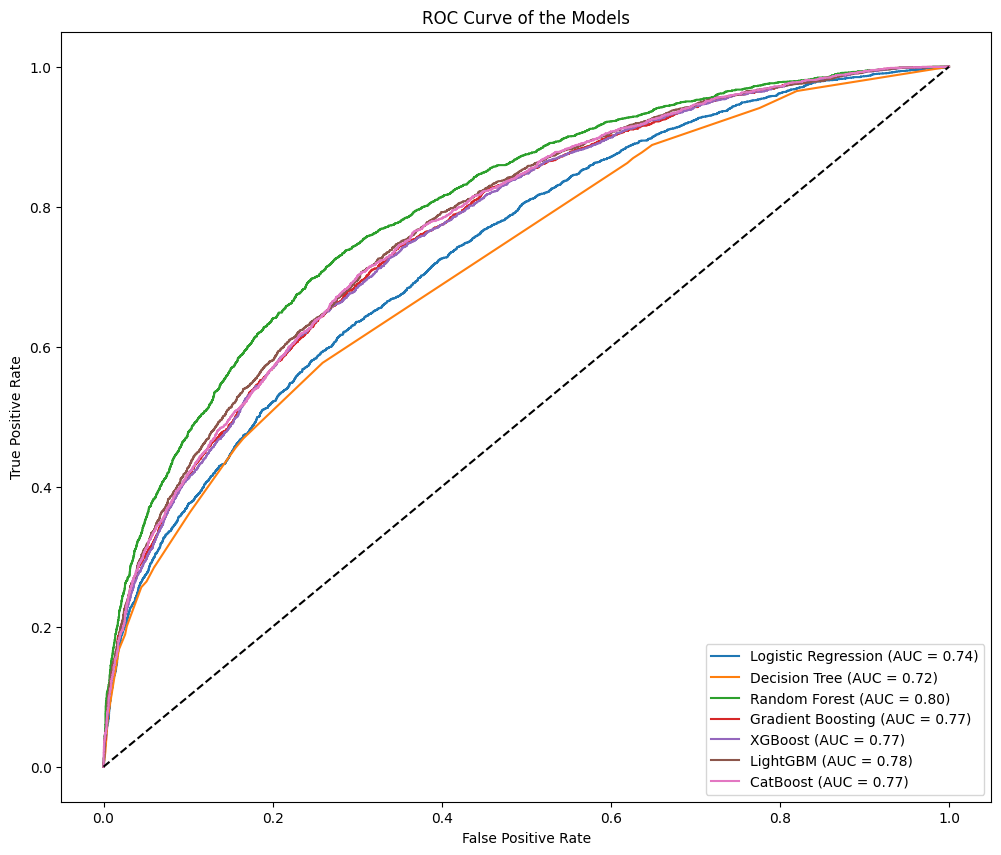


**Supplementary Figure 7**. Roc curve showing the AUC value in the model considering colorectal cancer-specific mortality in > 2 years


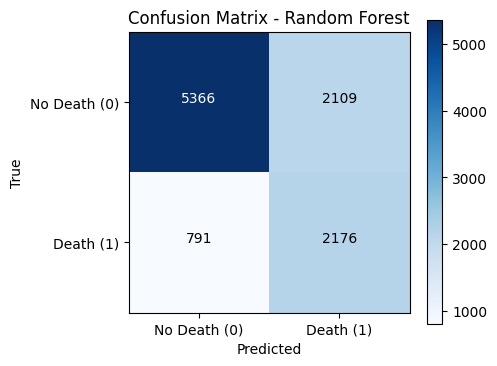


**Supplementary Figure 8**. Confusion matrix for Random Forest in the model considering colorectal cancer-specific mortality in > 2 years


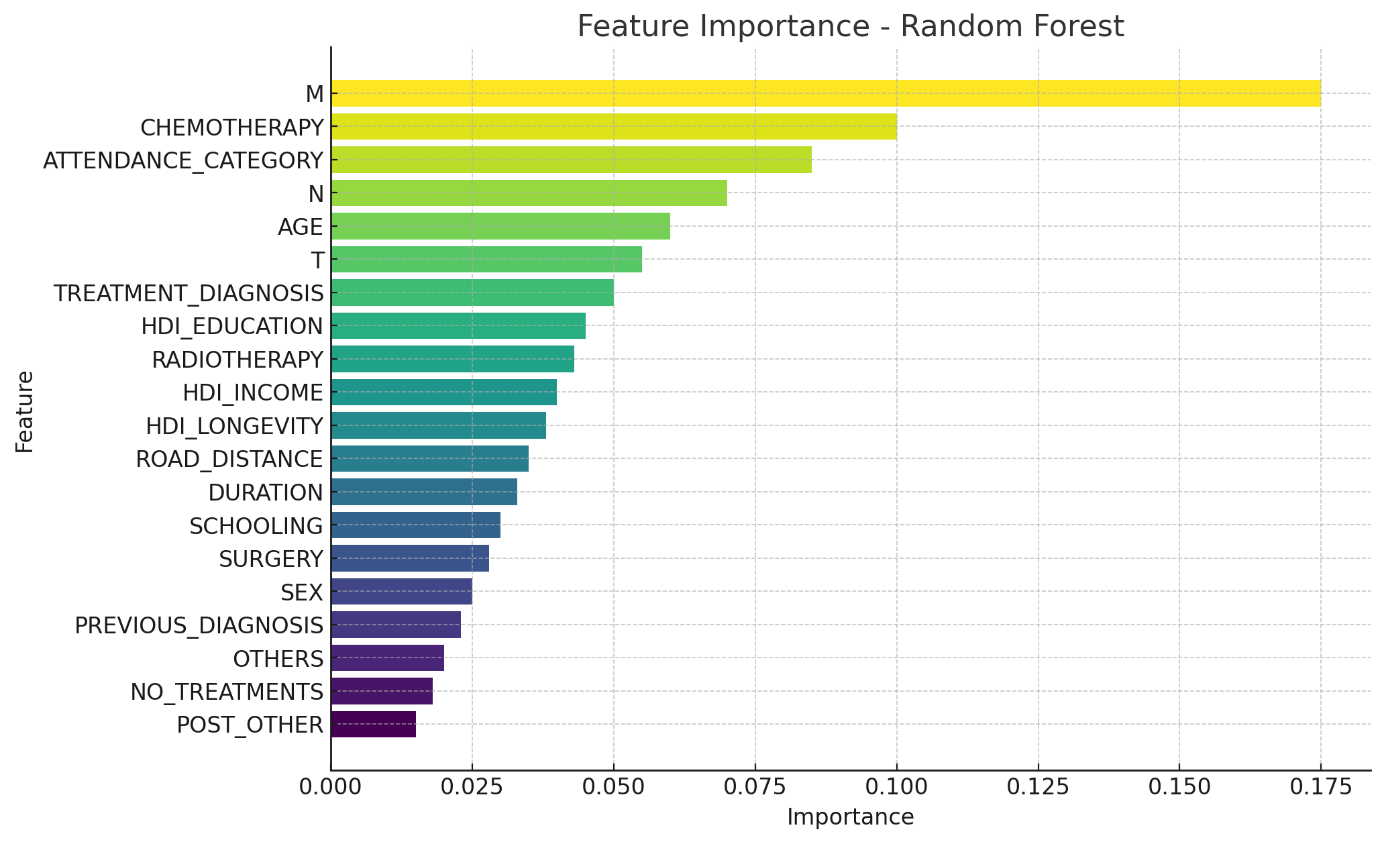


**Supplementary Figure 9**. SHAP graph showing the most important predictor variables in the model considering colorectal cancer-specific mortality in > 2 years

**Supplementary Material**

**Supplementary Table 1**. List of predictor variables used

| **Variable name** | **Description** | **Categories** | **Type** |
| --- | --- | --- | --- |
| **ORIGINAL DATABASE VARIABLES** | | | |
| Education | Education | Illiterate; incomplete elementary education; complete elementary education; secondary education; higher education; ignored | Categoric |
| Age | Age | - | Continuous |
| Sex | Sex | Male or female | Dichotomous |
| City | Patient's city of residence | Multiple categories | Categoric |
| Attendance_category | Service category | Health insurance, SUS, Private, no information | Categoric |
| Diagprev | Previous diagnosis and treatment | Without diagnosis / without treatment; with diagnosis / without treatment; with diagnosis / with treatment; others | Categoric |
| Ecgrup | Clinical staging group | - | Categoric |
| T | TNM classification - T | Date of diagnosis:  Up to 2005 – TNM 5th edition  2006 to 2013 – TNM 6th edition  From 2014 onwards – TNM 7th edition | Categoric |
| N | TNM classification - N | Date of diagnosis:  Up to 2005 – TNM 5th edition  2006 to 2013 – TNM 6th edition  From 2014 onwards – TNM 7th edition | Categoric |
| M | TNM classification - M | Date of diagnosis:  Up to 2005 – TNM 5th edition  2006 to 2013 – TNM 6th edition  From 2014 onwards – TNM 7th edition | Categoric |
| TMO | Treatment received in hospital = tmo | No or Yes | Dichotomous |
| Surgery | Treatment received in hospital = surgery | No or Yes | Dichotomous |
| Radio | Treatment received in hospital = radiotherapy | No or Yes | Dichotomous |
| Chemo | Treatment received in hospital = chemotherapy | No or Yes | Dichotomous |
| Hormone | Treatment received in hospital = hormone therapy | No or Yes | Dichotomous |
| Immuno | Treatment received in hospital = immunotherapy | No or Yes | Dichotomous |
| Others | Treatment received in hospital = others | No or Yes | Dichotomous |
| Nonepos | Treatment received in hospital = nonepos | No or Yes | Dichotomous |
| Surgerypos | Treatment received outside the hospital and during/after admission = surgery | No or Yes | Dichotomous |
| Radiopos | Treatment received outside the hospital and during/after admission = radiotherapy | No or Yes | Dichotomous |
| Chemopos | Treatment received outside the hospital and during/after admission = Chemotherapy | No or Yes | Dichotomous |
| Hormonepos | Treatment received outside the hospital and during/after admission = hormone therapy | No or Yes | Dichotomous |
| Immunopos | Treatment received outside the hospital and during/after admission = immunotherapy | No or Yes | Dichotomous |
| Otherspos | Treatment received outside the hospital and during/after admission = others | No or Yes | Dichotomous |
| Diagtrat | Difference in days between treatment and diagnosis dates | - | Continuous |
| **VARIABLES COLLECTED AND INSERTED INTO THE DATABASE** | | | |
| HDI_education | HDI Education Index | - | Continuous |
| HDI_income | HDI Income Index | - | Continuous |
| HDI_Longevity | HDI Longevity Index | - | Continuous |
| Road_distance | Road distance from the place of residence to the place where the patient is being treated | - | Continuous |
| Duration | Duration it takes for the patient to travel from their place of residence to the place of care | - | Continuous |

**Supplementary Table 2**. Comparative performance metrics of Random Forest models with and without socioeconomic variables

| **Metric** | **Clinical + socioeconomic** | **Clinical only** |
| --- | --- | --- |
| AUC-ROC | 0.92 | 0.79 |
| Accuracy | 0.85 | 0.69 |
| Precision | 0.83 | 0.62 |
| Sensitivity (Recall) | 0.82 | 0.79 |
| F1-Score | 0.83 | 0.69 |
| Specificity | 0.87 | 0.80 |

**Supplementary Table 3**. Final hyperparameters of the machine learning models

| **Model** | **Hyperparameter** | **Final value** |
| --- | --- | --- |
| **Logistic Regression** | C | 100 |
|  | Penalty | L2 (default) |
|  | Solver | liblinear / lbfgs (default) |
|  | Max iterations | 1000 |
| **Decision Tree** | Max depth | 10 |
|  | Min samples split | 10 |
|  | Min samples leaf | 4 |
| **Random Forest** | Number of trees (n_estimators) | 100 |
|  | Max depth | 20 |
|  | Min samples split | 5 |
|  | Bootstrap | True (default) |
| **Gradient Boosting** | Number of estimators | 100 |
|  | Learning rate | 0.10 |
|  | Max depth | 5 |
| **XGBoost** | Number of estimators | 100 |
|  | Learning rate | 0.20 |
|  | Max depth | 5 |
|  | Objective | binary:logistic |
| **LightGBM** | Number of estimators | 100 |
|  | Learning rate | 0.10 |
|  | Max depth | 10 |
| **CatBoost** | Iterations | 200 |
|  | Learning rate | 0.20 |
|  | Depth | 5 |
|  | Loss function | Logloss |

**Note**: All hyperparameters were selected via randomized cross-validation optimizing AUC-ROC.
